# Editorial: Computational solutions for microbiome and metagenomics sequencing analyses, Volume II

**DOI:** 10.3389/fmolb.2023.1253303

**Published:** 2023-07-17

**Authors:** Jovial Niyogisubizo, Yunpeng Cai, Lu Zhang, Xingyu Zhang, Yanjie Wei

**Affiliations:** ^1^ Centre for High Performance Computing, Joint Engineering Research Center for Health Big Data Intelligent Analysis Technology, Shenzhen Institutes of Advanced Technology, Chinese Academy of Sciences, Shenzhen, China; ^2^ Shenzhen Institutes of Advanced Technology, Chinese Academy of Sciences (CAS), Shenzhen, China; ^3^ Department of Computer Science, Hong Kong Baptist University, Kowloon, Hong Kong SAR, China; ^4^ School of Medicine, University of Pittsburgh, Pittsburgh, PA, United States

**Keywords:** gut microbiota, circular RNA biomarkers, metagenomics sequencing, COVID-19, feature selection, machine learning, immune markers

Microorganisms make up more than 17% of the known biomass on Earth and exhibit tremendous biodiversity. Microorganisms offer a vast Research Topic of genetic material for bioscience research, as the human microbiota alone contains 300 times more genes than the human genome. These genes play a crucial role in various essential biological functions. Investigating gene function is a key objective of microbiome genomics. Recognition of the pervasive influence of microbiota on ecosystems and human health has been limited by the challenges of detecting and culturing them using conventional methods. However, advancements in high-throughput sequencing technologies have led to a growing understanding of their importance.

Microbes are central to the biology of various living organisms, namely, humans, animals, and plants. The collection of microbiome sequence data has become more accessible and affordable, leading to a significant rise in the amount of data available for research. However, this surge in data has created a pressing demand for advanced computing resources to effectively analyze these massive datasets. Despite the establishment of a fair number of computational pipelines and numerous bioscience breakthroughs in recent years, the tremendous diversity and fast-evolving nature of microorganisms still pose severe challenges to the accurate and reliable resolution of metagenomic sequencing data. The aim of this Research Topic, “Computational Solutions for Microbiome and Metagenomics Sequencing Analyses, Volume II” is to address the computational challenges posed by the processing of large-scale microbial metagenomic sequencing data in single or multiple aspects such as taxonomic binning, functional characterization, biomarker discovery, and community dynamic modeling. This Research Topic contains five articles representing the latest advances in microbiota studies, genomic reconstruction, methylation-based understanding of COVID-19 expression, and circular RNA (circRNA) biomarkers for gastric cancer (GC) identification.

Gut microbiota Gd-IgA1-associated enzymes are involved in IgA nephropathy. The systematic metagenomics-based analysis conducted by Liang et al. revealed significant associations between specific gut microbiota and Gd-IgA1-associated enzymes in IgA nephropathy (IgAN). Pathway analysis indicated the involvement of microbiota-related metabolic pathways, while microbial enzyme analysis highlighted the enrichment of Gd-IgA1-associated α-galactosidase and α-N-acetyl-galactosaminidase secreted by Flavonifractor plautii in IgAN patients. These findings suggest a potential role for abnormal intestinal microbiota and their enzymes in Gd-IgA1 production and the pathogenesis of IgAN.

Understanding genomic mechanisms is the key to unraveling the intricate workings of genes. Frolova et al. conducted a comprehensive study on the synthesis of short-chain fatty acids (SCFAs) by the human gut microbiota (HGM). The authors reconstructed metabolic pathways for SCFAs and lactate synthesis in 2,856 bacterial genomes, representing over 800 known HGM species. By classifying genomes based on their ability to produce SCFAs, they assessed the SCFA synthesis potential in different HGM samples. The results show that the Research Topic of SCFA pathway genes and phenotypes enables predictive metabolic profiling and facilitates the study of gut microbiome interactions.

Two articles on this Research Topic utilized machine learning (ML) methods to identify specific markers related to COVID-19. Li et al. focused on the analysis of methylation data and employed feature selection and decision tree algorithms to extract key biomarkers and classification rules that effectively distinguish COVID-19 patients. The findings suggest that this method can discriminate COVID-19 at the methylation level, guiding diagnosis and treatment. Li et al. investigated the identification of COVID-19-specific immune markers using ML. The study highlights the close relationship between severe acute respiratory syndrome coronavirus 2 (SARS-CoV-2) and the immune system. It identifies cell markers through single-cell profiling and feature selection methods that differentiate COVID-19 from other respiratory diseases and healthy populations. providing insights into COVID-19 pathogenesis and potential intervention strategies.

Gastric cancer is a leading cause of mortality and does not have an ideal biomarker for its diagnosis and treatment. This hinders early detection and highlights the need for effective markers to improve patient outcomes. Hossain et al. reported that circRNAs show promise as a stable and potentially informative biomarker for GC diagnosis. The study used next-generation sequencing to identify differentially expressed circRNAs in GC samples, and the analysis revealed that circRNAs that interact with GC-related miRNAs are associated with important therapeutic and prognostic markers for GC.

To explore this Research Topic, the first article developed a promising approach using next-generation sequencing to identify circRNAs as potential biomarkers for GC. Other articles proposed ML methods to identify methylation-based biomarkers for COVID-19, distinguish COVID-19 from other respiratory diseases using immune markers, understand the genomic mechanisms behind short-chain fatty acid production by the human gut microbiota, and understand the role of gut microbiota enzymes in IgA nephropathy. All of these findings demonstrate the use of advanced computational techniques for microbiome and metagenomics sequencing analyses. We hope that this Research Topic will provide a comprehensive understanding of the computational challenges and potential solutions for future advancements in the field of microbiome genomics and metagenomics sequencing analyses.

